# Honokiol and Magnolol as Multifunctional Antioxidative Molecules for Dermatologic Disorders

**DOI:** 10.3390/molecules15096452

**Published:** 2010-09-16

**Authors:** Jui-Lung Shen, Kee-Ming Man, Po-Hsun Huang, Wen-Chi Chen, Der-Cherng Chen, Ya-Wen Cheng, Po-Len Liu, Ming-Chih Chou, Yung-Hsiang Chen

**Affiliations:** 1 Institute of Medicine, Chung Shan Medical University, Taichung; Taiwan; E-Mails: shen5300@yahoo.com.tw (J.L.S.); yw0727@mail2000.com.tw (Y.W.C.); 2 Department of Anesthesiology, Tungs’ Taichung MetroHarbor Hospital, Taichung; Taiwan; E-Mail: man_jimmy60@hotmail.com (K.M.M.); 3 Department of Dermatology, Taichung Veterans General Hospital, Taichung; Taiwan; 4 Graduate Institute of Integrated Medicine, College of Chinese Medicine, Graduate Institute of Clinical Medical Science, Department of Urology, Department of Neurosurgery, China Medical University and Hospital, Taichung; Taiwan; E-Mails: wgchen@mail.cmu.edu.tw (W.C.C.); vincenchen1966@gmail.com (D.C.C.); yhchen@mail.cmu.edu.tw (Y.H.C.); 5 Graduate Institute of Geriatric Medicine, Anhui Medical University, Hefei; China; 6 Division of Cardiology, Taipei Veterans General Hospital, Institute of Clinical Medicine, Cardiovascular Research Center, National Yang-Ming University, Taipei; Taiwan; E-Mail: huangbsvgh@gmail.com; 7 Department of Respiratory Therapy, College of Medicine, Kaohsiung Medical University, Kaohsiung; Taiwan; E-Mail: kisa@kmu.edu.tw

**Keywords:** antibacterial, antioxidant, Chinese medicine, dermatology, honokiol, inflammation, magnolol

## Abstract

Chinese herbs have been and still are widely used as important remedies in Oriental medicine. Over the recent years, a variety of biologically active constituents have been isolated from these sources and confirmed to have multifunctional activity in experimental studies. Honokiol is a small-molecule polyphenol isolated from the genus *Magnolia*. It is accompanied by other related polyphenols, including magnolol, with which it shares certain biological properties. Recently, honokiol and magnolol have been found to have anti-oxidative, anti-inflammatory, anti-tumor, and anti-microbial properties in preclinical models, without appreciable toxicity. These findings have increased interest in bringing honokiol and magnolol to the clinic as novel therapeutic agents in dermatology. In this review, the findings concerning the major mechanisms of action of honokiol and magnolol are described. Knowledge of the multiple activities of honokiol and magnolol can assist with the development of honokiol and magnolol derivatives and the design of clinical trials that will maximize the potential benefit of honokiol and magnolol in the patient setting for dermatologic disorders.

## 1. Introduction

Herbal therapy is becoming increasingly popular among physicians and patients [1,2]. Many medical plant preparations are marketed to the public for various ailments, including those of the skin [3,4,5]. Herbal therapies have been used successfully in Asia and Europe for treating dermatologic disorders (such as acne, wounds and burns, bacterial and fungal infections, dermatitis and psoriasis, and skin tumors) for thousands of years. In Asia, herbal treatments that have been used for centuries are now being studied scientifically. In United States, the herbal products are considered as dietary supplements or cosmetic additives. Since there is no standardization of active ingredients, purity, or concentration, this has made learning about and using these treatments challenging [6].

**Figure 1:**
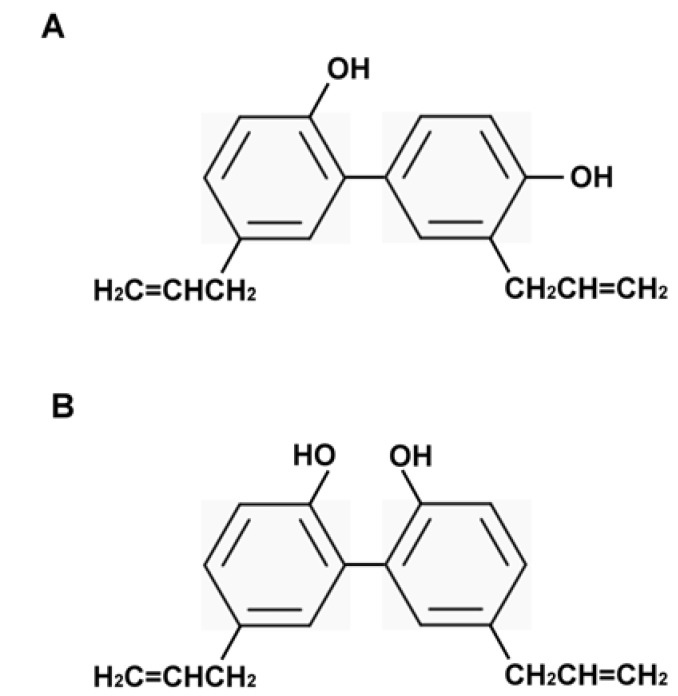
The chemical structures of (A) honokiol and (B) magnolol.

**Figure 2:**
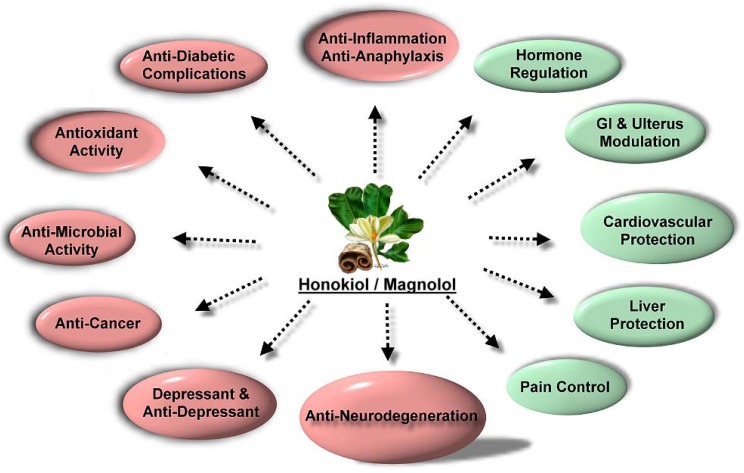
The flower, leaf, and bark of traditional Chinese medicinal plant *Magnolia officinalis* and the medicinal properties of honokiol/magnolol.

Honokiol and magnolol (Figure 1) were initially described as components of the genus *Magnolia*, which are components of Chinese (Kampo) herbs, including houpo and saiboku-tu(o) [7]. In the 1990s, honokiol and magnolol were found to have activity as free radical and lipid peroxidation inhibitors [8,9,10,11]. Thereafter, honokiol, magnolol, and a methanolic extract of *Magnolia* were shown to exhibit antioxidative, anti-inflammatory, anti-tumor, anti-diabetic complications, anti-microbial, anti-neurodegeneration, anti-depressant, pain control, hormone, gastrointestinal, and uterus modulation, cardiovascular and liver protective properties (Figure 2). The focus of this review are the recent findings regarding the biological effects of the antioxidative molecules honokiol and magnolol in dermatology (Table 1). Knowledge of the multiple biological activities of honokiol and magnolol could assist with the development of honokiol and magnolol derivatives and the design of clinical trials that will maximize the potential benefit of honokiol and magnolol in the patient setting for dermatologic disorders [12,13].

## 2. General Antioxidative Effects

This inhibition of oxidative stress was demonstrated *in vitro*; honokiol and magnolol reduce free radicals that generated by ultra-violet (UV) irradiation and inhibit UV-induced mutation in *Salmonella* [10]. This inhibition of lipid peroxidation was then *ex vivo* demonstrated in rat heart/liver mitochondria and human sperm [11,14]. It is approximately 1,000 times more potent than α-tocopherol [11,15] in inhibiting lipid peroxidation in heart mitochondria and 340 times more potent in rat liver mitochondria [14]. In the *in vivo* animal studies, magnolol protects against small intestinal, cerebral, and hind limb ischemia-reperfusion injury [16,17,18,19].

The *in vitro* cell culture system reveals the possible underlying mechanisms that honokiol and/or magnolol inhibit xanthine oxidase [20], protects mitochondrial respiratory chain enzyme, attenuates extracellular signal-regulated kinase (ERK) activation, and suppresses protein kinase C (PKC) and NADPH oxidase activities, resulting in the neutrophil respiratory burst inhibition [21] and cellular protection [17,22]. Dikalov *et al.* studied the reactions of honokiol in cell-free and cellular systems using electron spin resonance (ESR) and high-performance liquid chromatography (HPLC) techniques suggesting that honokiol is an effective scavenger of free radicals [23]. Additionally, the polyphenols may prevent formation of hydroxyl radical by chelating the transition metals such as copper and iron or repair molecules after free radical attack.

**Table 1:** Anti-oxidative, anti-inflammatory, anti-tumor, and anti-microbial effects and related mechanisms/outcomes of honokiol and/or magnolol.

Biological Effect	Mechanism / Outcome	Reference
**I. General Antioxidant Activity**		
Protection of heart mitochondria against lipid peroxidation	Free radical scavenging activity	[11]
Inhibition of xanthine oxidase	Antioxidative function	[20]
Inhibition of UV-induced mutation in *S. typhimurium*	Scavenger of free radicals generated by UV irradiation	[10]
Protection of sperm motility	Inhibition of lipid peroxidation	[73]
Protection of biological systems and functions	Protection of red cells and mitochondrial respiratory chain enzyme activity; against NADPH-induced peroxidative stress	[22]
Inhibition of fMLP-induced respiratory burst in neutrophils	Attenuation of ERK activation, and suppression of PKC and NADPH oxidase activities	[21]
Protective effect on the small intestinalI/R injury	Antioxidative function	[18,19]
Against heatstroke reactions	Against cerebral ischemic injury by antioxidative activity	[16]
Against cell killing, DNA damage, and lipid peroxidation	Antioxidative function	[17]
Protective efficacy in hind limb ischemia-reperfusion injury	Antioxidant, anti-nitrosative, and anti-inflammatory actions	[74]
Effective scavenger of ROS	Potent scavenger of free radicals	[23]
**II. Anti-Inflammation and Anti-Tumor**		
Inhibitory effects on mouse skin tumor promotion	Inhibition of EBV-EA activation induced by TPA	[28]
Anti-inflammatory and analgesic effects	Decrease of myeloperoxidase activity	[30]
Inhibition of plasma leakage in passive cutaneous anaphylactic reaction, neurogenic inflammation, dorsal skin and ear edema	Nonselective inhibition on vascular tissue to prevent the permeability change caused by various mediators	[44]
Inhibition of A23187-induced pleurisy	Reduction of eicosanoids mediator formation in the inflammatory site; suppression of PAF production in PMNs	[32,33]
Inhibition of the reduction of phorbol ester-induced neutrophil aggregation	Suppression of PKC activity; induction of cytosolic-free Ca^2+^ elevated neutrophils via IP3 signaling pathway	[46,47]
Inhibition macrophage activation	Inhibition of NO and TNF-α production in LPS-activated macrophages	[42]
Clinical efficacy in patients with asthma	Suppression of LTC4 release; inhibition of leukocyte leukotriene release; inhibition of 11β-hydroxysteroid dehydrogenase	[34,35,36,37,38,39]
Inhibition NO production in LPS-activated macrophages	Inhibition of NF-κB activation	[48]
Against allergy and anaphylaxis	Anti-histamine release on mast cells; inhibition of PLA2, 5-LO, LTC4 synthase and LTA4 hydrolase which are essential for LT-synthesis	[37,40]
Decrease in the excitability of airway myocytes	Stimulation of BK_Ca_ channel activity in tracheal smooth muscle cells	[75]
Inhibition of smooth muscle contraction in trachea	Blockade of Ca^2+^ influx through voltage-operated Ca^2+^ channels instead of Ca^2+^ release from intracellular Ca^2+^ stores	[76]
Influence of eicosanoid metabolism in neutrophils	Inhibition of prostaglandin and leukotriene formation	[31]
Early protection against endotoxin challenge (following sub-lethal hemorrhage)	Alteration of the course of endotoxin tolerance and cytokine response; attenuation of peroxidative damage	[53,54,55]
Anti-inflammatory effect of on neutrophils	Inhibition of ROS production	[29]
Inhibitory effect on tumor metastasis	Ability to inhibit tumor cell invasion	[51]
Prevention of skin photoaging	Inhibition of bFGF and MMP-1	[43]
Against passive cutaneous anaphylaxis reaction and scratching behaviors	Inhibition of IL-4 and TNF-α	[41]
Inhibition of various inflammatory events mediated by monocytes/macrophages and lymphocytes	Suppression of PI3K/Akt pathway	[45]
Suppression of NF-κB activation and NF-κB regulated gene expression	Inhibition of IκB kinase activation	[49]
Inhibition of proliferation of malignant melanoma cells	Activation of both mitochondrial and death receptor pathways	[52]
Chemopreventive effects on UVB-induced skin cancer development	Activating pro-apoptotic proteins through both intrinsic and extrinsic pathways	[50]
**III. Anti-Microbial Activity**		
Antifungal Activity	Against *T. mentagrophytes*, *M. gypseum*, *E. floccosum*, *A. niger*, *C. neoformans*, and *C. albicans*	[56,57]
Acne-mitigating Activity	Against *Propionibacterium* sp. and reduces secretion of IL-8 and TNF-α induced by *P. acnes* in THP-1 cells; inhibition of downstream pathway of MEKK-1 in NF-κB activation signaling	[59,60]
Antibacterial Activity	Bactericidal against VRE and MRSA; Against *A. actinomycetemcomitans*, *P. gingivalis*, *P. intermedia*, *M. luteus*, and *B. subtilis*; anti-*H. pylori* activity	[57,58,77,78]
Synthesis and microbiological evaluation of honokiol derivatives as new antimicrobial agents	Honokiol-glycine showed improved water solubility and antibacterial activities against *E. coli* and *P. aeruginosa*	[61]

## 3. Anti-Inflammation and Anti-Tumor Effects

Oxidative stress and inflammation [24] play important roles in skin tumor promotion [25]. Skin cancer is the most prevalent of all cancer types and its incidence is expected to increase substantially [26]. Chemoprevention involves the administration of chemical agents to prevent initiation, promotion and/or progression that occurs during neoplastic development [26,27]. Konoshima *et al.* were the first to test the *Magnolia officinalis* neolignans, honokiol and magnolol, for inhibition of Epstein-Barr *virus* early antigen (EBV-EA) activation induced by 12-*O*-tetradecanoylphorbol-13-acetate (TPA) and concluded these plant derivatives exhibited remarkable inhibitory effects on mouse skin tumor promotion in an *in vivo* carcinogenesis test [28]. Previous data showing blockade of inflammatory enzyme/cytokine production, nuclear factor (NF)-κB activation, and leukocyte activation suggest that honokiol and magnolol would have anti-inflammatory properties at clinically achievable concentrations [13]. 

Liou *et al.* found that honokiol inhibited PMA- or fMLP-induced reactive oxygen species (ROS) production by neutrophils by distinct mechanisms including: (i) honokiol diminished the activity of assembled-NADPH oxidase, a major reactive oxygen species producing enzyme in neutrophils; (ii) two other important enzymes for reactive oxygen species generation in neutrophils, *i.e.*, myeloperoxidase and cyclooxygenase, were also inhibited by honokiol; and (iii) honokiol enhanced glutathione (GSH) peroxidase activity [29]. 

Immunological functions of magnolol and honokiol are important because incidences of skin diseases are also connected to immunological abnormalities. Honokiol/magnolol exhibits leukocyte suppression, anti-inflammation, and analgesic effects via decreasing myeloperoxidase activity [30], eicosanoids mediator [31,32,33] and leukotriene [34,35,36,37,38,39] formation, histamine release [37,40], as well as nitric oxide (NO), tumor necrosis factor-α (TNF-α), basic fibroblast growth factor (bFGF), matrix metalloproteinase (MMP)-1, and interleukin (IL)-4 production [41,42,43]. Magnolol inhibits passive cutaneous anaphylactic reaction [41], skin photoaging [43], neurogenic inflammation, as well as dorsal skin and ear edema [44]. The intracellular signaling pathways involved in the immunomodulation include the suppression of the PI3K/Akt pathway [45], PKC [46,47], and redox-sensitive transcription factor NF-κB [48,49] activation, while the signal pathways and kinases upstream of IκB kinase (IKK) activation might be involved in the action of honokiol and magnolol [49]. 

Recent basic and clinical studies have implicated solar UV radiation in various skin diseases including premature aging and skin cancers. Chronic UV radiation exposure-induced skin disorders are caused by the excessive induction of inflammation, oxidative stress and DNA damage. The use of chemopreventive agents, such as plant polyphenols, to inhibit these events in UV-exposed skin is gaining attention [27]. More recently, in addition to chemically induced skin cancer development, Chilampalli *et al.* studied the chemopreventive effects of honokiol on UVB-induced skin tumor development in SKH-1 mice, a model relevant to humans, and to elucidate the possible role of apoptotic proteins involved in the prevention of skin tumor development. The honokiol-pretreated group exhibited significant reduction in tumor multiplicity as compared to the control group. Mechanistic studies showed the possible involvement of caspase-3, caspase-8, caspase-9, poly (ADP-ribose) polymerase (PARP) and p53 activation leading to the induction of DNA fragmentation and apoptosis. Their results show that honokiol acted as a potential chemopreventive agent to prevent UVB-induced skin cancer development, possibly by activating pro-apoptotic proteins through both intrinsic and extrinsic pathways [50]. These results suggested that honokiol and magnolol may favorably supplement sunscreen protection, and may be useful for skin diseases associated with solar UV radiation-induced inflammation, oxidative stress, and DNA damage.

Ikeda *et al.* investigated the anti-metastatic effect of magnolol on tumor metastasis *in vivo* with experimental and spontaneous metastasis models with an experimental and spontaneous lung metastasis model using melanoma to clarify the mechanism [51]. In addition, magnolol inhibited proliferation of human malignant melanoma cells. It induced oligonucleosomal fragmentation of DNA in melanoma cells and increased caspase-3, 8, 9 activities followed by the degradation of caspase-3 substrates, inhibitor of caspase dependent DNase (ICAD) and PARP indicating that magnolol induces apoptosis by activation of both mitochondrial and death receptor pathways in melanoma cells [52]. These data from the *in vivo* and *in vitro* experiments suggest that magnolol possesses strong anti-metastatic and tumor suppressing ability and that it may be a lead compound for drug development. 

Additionally, magnolol provides early protection against endotoxin challenge (following sub-lethal hemorrhage) by altering the course of endotoxin tolerance and cytokine response and attenuating peroxidative damage [53,54,55]. These findings have increased interest in bringing magnolol to the clinic as a novel anti-inflammatory and anti-anaphylaxis agent for inflammation. Preparations containing magnolol have also been used in pilot clinical trials for inflammation-related disorders [34,35,36,38,39].

## 4. Anti-Microbial Effects

Clark *et al.* first tested the significant anti-microbial activity of magnolol using an agar well diffusion assay and found that honokiol and magnolol exhibited significant activity against Gram-positive and acid-fast bacteria and fungi [56,57]. The extract of *Magnolia officinalis* has been found to potently inhibit the growth of *Helicobacter pylori* [58]. *Propionibacterium acnes*, an anaerobic pathogen, plays an important role in the pathogenesis of acne and seems to initiate the inflammatory process by producing proinflammatory cytokines. Since magnolol had been known to exhibit antibacterial activities, Park *et al.* tested its antibacterial activity against *Propionibacterium sp.* In addition, they found the reduced secretion of IL-8 and TNF-α induced by *P. acnes* in THP-1 cells indicating the anti-inflammatory effects of them [59]. Together with the previously known antibacterial activity against *P. acnes* and based on these results, it is suggested that magnolol may be introduced as possible acne-mitigating agents [60].

More recently, to improve the solubility and antibacterial activity of honokiol against *E. coli *and *P. aeruginosa*, new honokiol-derivatives (honokiol acetate, honokiol succinic acid, honokiol glycerol, honokiol glycine, honokiol glucose and honokiol mannose) were synthesized and their solubility and antimicrobial activities were investigated by Kim *et al.* They reported that among the tested compounds, honokiol glycine showed improved water solubility and antibacterial activities against *E. coli* and *P. aeruginosa* when compared to honokiol [61].

## 5. Safety of Herbal Preparations

There are many herbal therapies available for dermatological diseases that patients have already begun to discover [62,63]. Many patients have the misconception that these have no adverse effects because herbs are “natural” [64]. However, herbal preparations vary greatly in their therapeutic indexes [65]. For example, some are consumed as foods and have high therapeutic indexes, and others are highly biologically active and must be used very carefully [66]. Dermatologists must be educated not only in the benefits of these therapies, but must also be aware of some of the risks and potential adverse effects [67]. They need information about the effects of herbal remedies in order to better serve their patients who may be using herbs to treat their dermatological conditions [6]. In addition to the adverse effects, patients should be counseled on the lack of regulation for herbal medicines. The design of clinical trials should be encouraged to maximize the potential benefit of honokiol and magnolol in the patient setting for dermatologic disorders. Furthermore, quality-control measures to ensure the purity [68,69], concentration [70], or safety of herbal supplements should be standardized scientifically based on evidence-base medicine data [71,72]. 

## 6. Conclusions

Honokiol and magnolol have shown strong antioxidative, anti-inflammatory, anti-tumor, and anti-microbial properties mediated by several modes of action. Thus, honokiol and magnolol exhibit a desirable spectrum of bioavailability rather than other natural products. To fully realize the potential of honokiol and magnolol, clinical trials are needed. Honokiol and magnolol analogues with improved pharmacokinetic and pharmacodynamics will also make the field move forward. Safety during long-term administration, combined with its cost and future therapeutic potential, makes it an ideal agent for both prevention and therapy in dermatology either alone or in combination with other drugs. This knowledge is required for the development of future analogues, which may target either of these pathways, and for the development of clinical trials using honokiol and magnolol or their analogues. Further insights into the signaling network and interaction points modulated by honokiol and magnolol may provide the basis for novel discovery programs to exploit honokiol and magnolol for the prevention and treatment of dermatologic disorders. 

**Figure 3:**
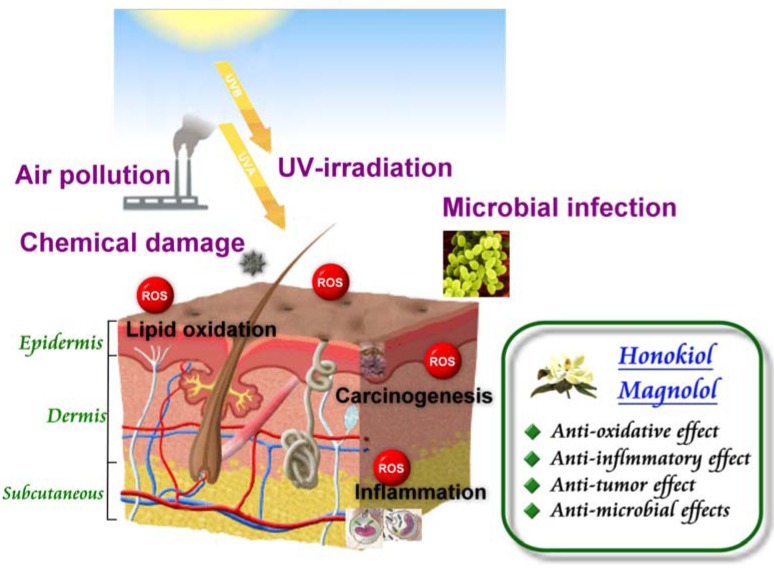
The protective effects of honokiol/magnolol for dermatologic disorders.
